# Neurotoxicity of Acrylamide in Exposed Workers

**DOI:** 10.3390/ijerph10093843

**Published:** 2013-08-27

**Authors:** Manuela Pennisi, Giulia Malaguarnera, Valentina Puglisi, Luisa Vinciguerra, Marco Vacante, Mariano Malaguarnera

**Affiliations:** 1 Department of Chemistry, University of Catania, Catania 95123, Italy; E-Mail: manuelapennisi@libero.it; 2 International Ph.D. Programme in Neuropharmacology, University of Catania, Catania 95123, Italy; 3 Department of Neurosciences, University of Catania, Catania 95123, Italy; E-Mails: valentinapuglisi@hotmail.com (V.P.); luisavinciguerra@hotmail.it (L.V.); 4 Department of Senescence, Urological, and Neurological Sciences, University of Catania, Catania 95126, Italy; E-Mails: marcovacante@yahoo.it (M.V.); malaguar@unict.it (M.M.)

**Keywords:** acrylamide, workers, neurotoxicity, neuropathy

## Abstract

Acrylamide (ACR) is a water-soluble chemical used in different industrial and laboratory processes. ACR monomer is neurotoxic in humans and laboratory animals. Subchronic exposure to this chemical causes neuropathies, hands and feet numbness, gait abnormalities, muscle weakness, ataxia, skin and in some cases, cerebellar alterations. ACR neurotoxicity involves mostly the peripheral but also the central nervous system, because of damage to the nerve terminal through membrane fusion mechanisms and tubulovescicular alterations. Nevertheless, the exact action mechanism is not completely elucidated. In this paper we have reviewed the current literature on its neurotoxicity connected to work-related ACR exposure. We have analyzed not only the different pathogenetic hypotheses focusing on possible neuropathological targets, but also the critical behavior of ACR poisoning. In addition we have evaluated the ACR-exposed workers case studies. Despite all the amount of work which have being carried out on this topic more studies are necessary to fully understand the pathogenetic mechanisms, in order to propose suitable therapies.

## 1. Introduction

Acrylamide (ACR) is an odorless crystalline solid at room temperature, with molecular formula C_3_H_5_NO and molecular weight of 71.08. The monomeric form of ACR is a water-soluble powder and it is employed in different chemical and industrial processes. It is a vinyl monomer and its industrial application is associated with pollution and health risks [1]. ACR is a component of polymers and co-polymer materials used in gel chromatography and in waste water management [2]. It was used in chemical grouts in order to avoid water seepage at construction, drilling, mining sites and manufacturing flocculators [3]. ACR is also a contaminant in foods prepared at very high temperatures. The report that acrylamide is formed during high-temperature cooking of animal feed and that is found in common human foods that are prepared by cooking at high temperatures has generated interest in the neurotoxicity of dietary exposure to acrylamide. Direct consumer exposure to acrylamide may result from ingestion of high-carbohydrate foods such as potato crisps and chips, roasted cereals, and breads. Indirect exposure may result from residual traces of the monomer in food packaging where polyacrylamide is used as a binding agent [4]. Several observations have led to the hypothesis that heating of food could be an important source of human exposure to acrylamide. Acrylamide is formed in foods, if the heating frying is done in a oven, on a frying pan or by microwave heating, whilst no acrylamide has been detected in boiled food products [5,6]. Even if the polymer is nontoxic, several studies showed that exposure of humans and laboratory animals to monomeric form of ACR causes neuropathies. Indeed, subchronic low-level work exposure to ACR may bring on ataxia, gait abnormalities, skeletal muscle weakness, skin abnormalities, and numbness of hands and feet. Some toxicological studies suggested that acrylamide vapours irritate the eyes and the skin and cause paralysis of the cerebrospinal system [4,7,8]. In laboratory animals (such as rodents, Guinea pigs, rabbits, cats and dogs) repeated daily exposure to this chemical (0.5–50 mg/kg per day) leads to neurological signs resembling the kind of neurotoxicity observed in humans [9]. In 1994 the International Agency for Research on Cancer classified acrylamide as “potentially carcinogenic to humans” [10] and in 2001 the Scientific Committee on Toxicity Ecotoxicity and the Environment demonstrated its inherent toxicity properties: neurotoxicity, genotoxicity to both somatic and germ cells, carcinogenicity, mutagenicity and reproductive toxicity) [11]. On the other hand epidemiological studies regarding work-related exposure in humans show no correlation between the ACR exposure and the increased cancer risk [12,13].

## 2. Experimental Research and Pathogenesis

**Figure 1:**
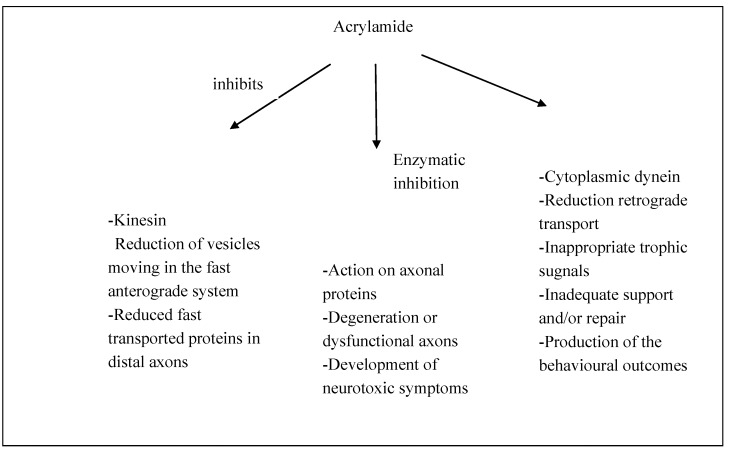
Model of ACR action leading to neurotoxicity.

Although evidence for neurotoxicity of chronic exposure to ACR from human studies is rather limited, it is fairly strong from animal studies especially those involving the immature brain [14,15,16]. In rodents ACR is metabolized and excreted in the urine as metabolites derived from reduced glutathione conjugation [17]. The toxic action mechanisms underlying the findings of these *in vitro* and *in vivo* studies are not yet well known. Several hypotheses have been suggested to explain the molecular mechanisms of neurotoxic action of the ACR, interaction with nucleic acids, second messenger systems, enzymes, receptors or translocating proteins, effects on neurotransmitter concentrations and reuptakes, disruption of membrane dynamics, and damage to glial cells resulting in accelerated lipoperoxidation that ultimately influences neuronal function. Various studies have demonstrated that exposure to monomeric ACR causes cellular damage in both the nervous and reproductive systems and produces tumors in hormonally responsive tissues [18]. Afterwards, some studies revealed that low dose subchronic ACR neurotoxicity was associated with nerve damage characterized by multifocal paranodal swelling of preterminal distal myelinated axons in both central and peripheral nervous systems [19]. These swellings contained a large amount of neurofilaments, tubulovescicular profiles and degenerating mithocondria. When the exposure to ACR continued, a progressive retrograde degeneration of these distal region of axons occurred, with preservation of proximal segments of axons [20,21]. Moreover these early studies hypothesized that sensory axons and their receptors were involved in this neuropathic process before autonomic and motor axons whereas other research studies showed that sensory, motor and autonomic axons were equally affected by the ACR neurotoxicity (or ACR-induced damage, Figure 1). Although the relative vulnerabilities of the sensory, autonomic and motor systems were debated, the pattern of neuropathological expression induced by ACR was consistent with the theory of toxic “dying-back” neuropathies proposed by Cavanagh [22,23]. According to this hypothesis, direct neurotoxic actions at cell body sites caused deficient production and transport of axon-directed components. The dying-back theory was supported by some following studies, suggesting that sensory and motor nerve cell body remodeling observed during intoxication was a direct neuropathogenic effect of ACR [24,25,26,27,28,29,30,31,32]. However, other studies suggested that dying-back theory did not completely explain the ACR-induced neuropathy. They hypothesized that degeneration did not occur at the nerve terminal at the beginning and that cell body involvement was a secondary effect of ACR intoxication [33,34,35,36]. Based on these assumptions, Schaumburg and Spencer (1976), proposed a theory that emphasized direct axonal injury. It suggested that large diameter axons in the Central Nervous System (CNS) and in the Peripheral Nervous System (PNS) were most sensitive to development of simultaneous multifocal paranodal axon swelling in distal regions, that gave rise to a subsequent degeneration. Historically, this type of nerve damage was defined “central-peripheral distal axonopathy” [37,38]. In recent years, Sickles *et al.* [39] hypothesized that ACR bound and inhibited the motor protein kinesin, leading to a dysfunction of bi-directional fast axonal transport. It was based on electrophysiological and pathological studies performed in laboratory animals. Lo Pachin [40] proposed that the primary site of structural and functional damage caused by ACR could be the nerve terminal in the PNS and CNS. ACR neurotoxicity could affect nerve terminal and could adduct cysteine residues on functionally important presynaptic proteins, involved in membrane-fusion processes. It results in neurotransmitter release inhibition and eventual degeneration. Other studies have shown that ACR could alter cytoskeletal proteins levels in rat sciatic nerve, as possible mechanism of perypheral neuropathy [41,42]. Recently, increasing evidence supports the notion that reduction of cellular expression and activity of antioxidant proteins and the resulting increase of oxidative stress are fundamental causes in many chemical induced cell damages [43]. Oxidative stress implies an increase in the generation of reactive oxigen species (ROS), that causes cellular damage through the attack of polyunsaturated fatty acids and lipid peroxidation of biomembranes. A recent study, performed by Zhu *et al.* [44], showed that decreased neural glutathione (GSH) could be one of the primary events in ACR-induced neurotoxicity. It is accepted that the neurotoxicity of acrylamide trial is cumulative and that the expression of axonal degeneration is exclusively related to the dose rate, *i.e.*, degeneration in the PNS and CNS is evident only during intoxication at lower ACR dose-rates. This indicates that the axonal degeneration during exposure to acrylamide is not a critical event in the pathophysiological process of neurotoxicity. The neurotoxicity of acrylamide is also expressed through the Purkinje cell injury, even if the mechanism of damage is not well known. It is thought to be due to tubulovescicular alterations or membrane-fusion mechanisms damage at the nerve terminal, as well as in the PNS. Oxidative stress and mitochondrial dysfunction have been demonstrated to be key mechanisms in many chemical-induced cell injuries and neurodegenerative diseases. Oxidative stress refers to enhanced generation of reactive species/reactive nitrogen species and/or depletion of antioxidant defence system causing an imbalance between pro-oxidants and antioxidants leading to apoptosis [45,46]. ACR enhanced the activity levels of acetylcholinesterase (AchE), a significant biological component of the cholinergic function and membrane, in both head and body regions [47].

## 3. Human Case Studies

Exposure mode changes depending on the type of work. The potential routes of human exposure to ACR are ingestion, dermal contact and inhalation. Another oral route of exposure is via drinking water contamined by polyacrylamide flocculants used in water treatment. Typically concerned workers relate to the construction industry, coal mine preparation plants (or mines), flocculator manufacturing, acrylamide manufacturing factories or tunnel construction. Contact with ACR occurs through hand skin and maybe by dust ingestion, due to handling of ACR monomer during polymerization process, or treatment of contaminated aqueous solution (Table 1).

**Table 1:** Acrylamide neurotoxicity case studies in literature.

Case Study	Patients	Type of Work	Exposition	Signs and Symptoms
Mulloy, K.B. 1996 [48]	2 workers in the Southern West Virginia	Coal mine preparation plant workers producing acrylamide monomers	Exposure for over 10 years to acrylamide polymer flocculent contaminated with acrylamide monomer	Patient A: parkinsonism Patient B: peripheral neuropathies, neurological bladder
Calleman, C.J. *et al.* 1994 [49]	41 workers in the city of Xinxiang	Workers heavily exposed to a mixture of acrylamide and acrylonitrile	Acrylamide and acrylonitrile	Peripheral neuropathy
Myers, J.E. *et al.* 1991 [50]	Workers at a South African factory	Factory workers producing polymer from acrylamide monomers	Acrylamide monomer	Peripheral neuropathy, abnormal gait, skin abnormalities
Auld, R.B. *et al.* 1967 [3]	A white male worker in Bathurst	Mine worker	10% Aqueous solution of acrylamide	Peripheral neuropathy
Kesson, C.M. *et al.* 1977 [51]	6 workers in England	Construction industry workers in the confines of a small tunnel	Acrylamide monomer	Peripheral neuropathy. Two workers affected more severely than the other ones
Garland, T.O. *et al.* 1967 [52]	6 workers at two English factories	Polymerization of the Acrylamide monomer in the manufacture of flocculators	Acrylamide monomer	Peripheral neuropathy, midbrain disturbance
He, F. *et al.* 1989 [53]	71 workers in China	Small factories manufacturing acrylamide workers	Aqueous solution of acrylamide monomer	Peripheral neuropathy, cerebellar involment (3 patients), skin abnormalities
Kjuus, H. *et al.* 2004 [54]	24 workers in Norway	Tunnel workers	N-methylolacrylamide (NMA)-containing grout and water contaminated with acrylamide	Peripheral neuropathy (paresthesia, pain and weakness in the limbs), skin abnormalities
Goffeng, L.O. *et al.* 2008 [55]	44 workers in Norway	Tunnel workers	N-methylolacrylamide (NMA)-containing grout and water contaminated with acrylamide	Slightly reduced light sensitivity and colour discrimination

The acrylamide-exposed workers showed high prevalences of paresthesia in hands and legs, cramp in legs, attacks of white fingers, skin irritation and peeling of skin on the hands, headache, and breathlessness [55,56,57,58]. The onset of symptoms occurred some weeks after the earlier contact. Usually the first pathological manifestations are: skin rash and hands (feet in some cases), sweating and peeling. Afterwards neurological signs become evident in the form of a peripheral neuropathy: muscle weakness and altered sensitivity of the extremities. Generally an impairment of position, temperature and vibration sensation are present. In some cases pain, paresthesias, loss of balance and truncal ataxia may appear. ACR-induced neurotoxicity diminished ATP-ase activity, enhanced activity of acetilcholinesterase and dopamine depletion. Neurotoxicity is characterized by ataxia and skeletal muscle weakness. Numbness in hands and feet, fatigue and sweating of hands and feet are prominent symptoms of ACR intoxication [59]. Neuropathological studies suggested that ACR neurotoxicity was mediated by distal axon degeneration. Only a few patients complained tremors, slurring of speech, nystagmus, clumsiness, bladder disturbance, lethargy, cramps and dizzy. Slightly reduced light sensitivity and color discrimination could appear. Neurological examination showed in some patients tendon reflexes loss, muscle wasting of extremities and positive Romberg sign. ACR-induced neurological symptoms that initially developed as hindlimb muscle weakness and ataxia were described in 1967 Garland and Patterson in six workers at two factories [52] and Auld and Baldwell in a white mine worker [3]. ACR-induced neuropathy has been classified either as a central peripheral distal axonopathy, or sensory-motor distal symmetrical polyneuropathy with neurofilamentous axonopathy [48,51,59].

In acrylamide-exposed tunnel workers Kjuus *et al.* [54] examined 2–10 years post-exposure, observed a reduced sural sensory nerve conduction velocity, indicating a possible persistent effect on the peripheral nervous system. Furthermore, Goffeng *et al.* [55] reported reduced visual system function related to color vision and light sensitivity, among these tunnel workers, indicating persisting axonal visual system effects. Visual system effect following acrylamide exposure have been reported in experimental animal studies and also in a human case report [60]. In cattle after accidental acrylamide intoxication, Godin *et al.* [61] observed abnormal papillary light reflex, progressive retinal degeneration and changes in optic nerve discs, indicating degeneration of optic nerve neurons. Electroneuromyographic studies evidenced prolonged duration of motor unit potential and increased polyphasic potential even in workers with no neuropathy signs. Furthermore, sensory action potentials is significantly decreased. After stopping the exposure to the chemical, only the mildly affected patients had a complete recovery [51].

## 4. Prevention and Health Risk Assessment

ACR toxic exposure prevention should aim to avoid inhalation, dermal uptake or ingestion. A good protection for the skin is given by long gloves, head covering, washable overalls and face shield. Light masks screen workers from flying dust. Periodic medical monitoring is necessary. Regarding occupational surveillance, hemoglobin adducts are useful *in vivo* dose indicators of exposure to ACR and possible predictors of ACR-induced peripheral neuropathy. Vibration threshold assessments could be helpful recognizing early ACR neurotoxic effects in workers [62].

## 5. Medical Treatment of ACR Neurotoxicity

Medical treatment in humans is symptomatic. The best therapy is stopping the exposure to the chemical. Nonetheless after a complete withdrawal from work, only the mildly affected workers underwent complete recovery [63]. Tareke *et al.* [64] showed that addiction of BHT, sesamol and vitamin E to meat prior the heating enhanced the formation of acrylamide, by protection of ACR against free radical-initiated reactions. Natural products and synthetic derivatives, such as dark soy, panax ginseng, tea polyphenols, vitamin B6, thioctic acid, l-carnitine and its derivatives, resveratrol, melatonin, protect against ACR dependent neurotoxicity via antioxidant mechanisms [65,66,67,68,69]. Levine and Smith [70] demonstrated that NaHSO_3_ (as preservative and antioxidant compound) enhances acrylamide elimination. Casado *et al.* [71] suggested that NaHSO_3_ may inhibit the production of intermediates that induce and then reduce the formation of ACR without a negative repercussion on the sensory quality of heated olive juice. Mustafa *et al.* [72] studied the effect of time and temperature of baking and addition of fructose, asparagine and oat bran concentrate on the acrylamide content and colour of rye crisp bread. Acrylamide content increased with time and temperature of baking, with higher effects perceived at higher temperatures and longer times in an accelerating slope. They showed that baking rye crisp bread at different combination of time and temperature favoured the browning reactions. There is a positive correlation between baking temperature and time and acrylamide formation whilst replacement of reducing sugars with sucrose and the use of flours, with a lower asparagine content may decrease the acrylamide content of baked foods [73].

## 6. Conclusions

ACR requires multiple exposure to produce neurotoxicity. Low-level, work-related exposure to ACR monomer is neurotoxic for humans. The ACR poisoning usually involved construction industry workers, mine workers, flocculator manufacture workers and tunnel workers. Whereas the exact mechanism of action of ACR is not completely clarified, it is shown that ACR neurotoxicity is cumulative. Short-term occupational exposure induced weak legs, loss of toe reflexes and sensations, and numb hands and feet preceded by skin peeling from the hands [50,53,74]. Longer exposure resulted in more severe symptoms including cerebellar dysfunction followed by neuropathy. Currently there are two mechanistic hypotheses of acrylamide neurotoxicity: inhibition of kinesin-based fast axonal transport and direct inhibition of neurotransmission [39]. Vitamin B6, thioctic acid, sodium pyruvate, and 4-methylcatechol have been shown to protect against or accelerate recovery from acrylamide induced neuropathy [49,75,76,77]. Further studies are necessary to clarify the pathogenesis and to improve the treatment.
